# Deficient Novelty Detection and Encoding in Early Alzheimer’s Disease: An ERP Study

**DOI:** 10.1007/s10548-022-00908-x

**Published:** 2022-08-20

**Authors:** Domilė Tautvydaitė, Alexandra Adam-Darqué, Paulina Andryszak, Léa Poitrine, Radek Ptak, Giovanni B. Frisoni, Armin Schnider

**Affiliations:** 1 Laboratory of Cognitive Neurorehabilitation, Division of Neurorehabilitation, Department of Clinical Neurosciences, University Hospital of Geneva and University of Geneva, Av. de Beau-Séjour 26, 1211 Geneva, Switzerland; 2 Laboratory of Neuroimaging of Aging (LANVIE), University of Geneva, Geneva, Switzerland; 3 Department of Rehabilitation and Geriatrics, Memory Clinic, Geneva University and University Hospitals, Geneva, Switzerland

**Keywords:** Alzheimer’s disease, Evoked potentials, Inverse solution, Medial temporal lobe, Memory encoding, Novelty detection

## Abstract

Patients with early Alzheimer’s disease (AD) have difficulty in learning new information and in detecting novel stimuli. The underlying physiological mechanisms are not well known. We investigated the electrophysiological correlates of the early (< 400 ms), automatic phase of novelty detection and encoding in AD. We used high-density EEG Queryin patients with early AD and healthy age-matched controls who performed a continuous recognition task (CRT) involving new stimuli (New), thought to provoke novelty detection and encoding, which were then repeated up to 4 consecutive times to produce over-familiarity with the stimuli. Stimuli then reappeared after 9–15 intervening items (N-back) to be re-encoded. AD patients had substantial difficulty in detecting novel stimuli and recognizing repeated ones. Main evoked potential differences between repeated and new stimuli emerged at 180–260 ms: neural source estimations in controls revealed more extended MTL activation for N-back stimuli and anterior temporal lobe activations for New stimuli compared to highly familiar repetitions. In contrast, AD patients exhibited no activation differences between the three stimulus types. In direct comparison, healthy subjects had significantly stronger MTL activation in response to New and N-back stimuli than AD patients. These results point to abnormally weak early MTL activity as a correlate of deficient novelty detection and encoding in early AD.

## Background

Patients with early Alzheimer’s disease (AD) have difficulty in learning new information, indicating impaired encoding (Peña-Casanova et al., 2012). This failure is explained by the early involvement of the medial temporal lobe (MTL) in the disease (Braak et al., 1999). However, patients also have false familiarity with stimuli that they have not seen before (Budson et al., 2000). While this difficulty might be explained by weak encoding of information, it may also reflect deficient detection of the novelty of stimuli (Budson et al., 2006). Though it is difficult to clearly separate encoding from novelty detection, the former is known to depend strongly on the depth of processing (e.g., recruitment of superficial, visual rather than semantic processes), which is not necessary for the latter. In the realm of memory, encoding and novelty detection are linked: healthy subjects encode novel stimuli better than familiar stimuli (Nyberg, 2005; Ranganath & Rainer, 2003; Tulving & Kroll, 1995). In addition, neuroimaging studies indicate that similar neural sources in the MTL are implicated in novelty detection and encoding (Grunwald & Kurthen, 2006; Ranganath & Rainer, 2003).

In AD patients, deficient novelty detection was demonstrated using oddball paradigms, which require detection of stimuli “popping out” from a series of stimuli (Knight, 1996). In healthy subjects, novelty detection in the oddball paradigm typically evokes a midline fronto-parietal potential peaking at around 300 ms, the P300 (Polich, 2007). This potential is attenuated and delayed in AD (Bennys et al., 2007; Hedges et al., 2016). However, the relevance of novelty processing in the oddball paradigm for memory encoding is unclear. Memory effects in healthy subjects are typically observed at later stages in form of an enhanced frontal positivity evoked by new stimuli at about 300–600 ms, also labelled the P600 (Addante et al., 2012; Curran & Cleary, 2003; Duarte et al., 2004; Friedman & Johnson, 2000; Hoppstädter et al., 2015; Rugg & Curran, 2007). In patients with AD or amnestic mild cognitive impairment (MCI; a precursor of AD), this response is attenuated or absent (Olichney et al., 2006; Yang et al., 2015).

Paradigms investigating old/new effects cannot unequivocally distinguish between the encoding of new stimuli as opposed to the recognition of old items. Raynal et al. (2020) described a continuous recognition paradigm comparing new pictures –reflecting novelty and encoding- with highly familiar stimuli repeated 4 consecutive times. In this study frontal positivity at 200–300 ms in response to novel items was estimated to emanate from the MTL. This result suggests that the processing of novelty –tested with an odd-ball paradigm- and encoding –tested with a dedicated continuous recognition task- may both occur at an early stage, around 200–300 ms. So far, such early processing has not been explored in AD, in particular not with respect to the novelty of stimuli in a memory paradigm.

In the present study we compared the early, automatic stage of encoding (< 400 ms) of novel as opposed to familiar stimuli in patients with early AD. In this context, “novelty detection” refers to the ability to detect a stimulus as new, rather than familiar from previous presentation; “encoding” refers to the process allowing for later recognition of a stimulus; finally, “re-encoding” refers to the additional encoding of stimuli already familiar from previous encounter. We composed a continuous recognition task (CRT) with 3 types of items differing in terms of novelty and incentive for encoding: (1) New designs evoking novelty detection and encoding; (2) stimuli repeated after 9–15 intervening stimuli, which are not novel but induce re-encoding, as reflected in better recognition after a delay (James et al., 2009.); (3) items repeated 3 to 4 times in immediate succession. As in Raynal et al. (2020), these highly familiar stimuli, unlikely to undergo relevant further encoding, were used as a “baseline” to seize encoding activity provoked by the stimuli (1) and (2). Our hypothesis was that, in healthy elderly subjects, the rapid, automatic processing of these stimuli in the first 400 ms after presentation induces evoked potential responses that vary as a function of stimulus novelty and incitement to encoding. In patients with early AD, signals reflecting novelty detection, encoding, or both would be attenuated or absent.

## Materials and Methods

### Participants

Twenty-nine patients (aged 72.7 ± 6.2 years) investigated for memory impairment at the Geneva University Hospital Memory Clinic, and 19 age-matched healthy controls (69 ± 7 years, 9 women) participated in the study. Five patients were excluded due to poor EEG signal and/or incapacity to perform the task, which made the final sample of 24 patients (72 ± 6 years, 14 women). All participants gave written informed consent to participate in the study, which was approved by the Ethical Committee of the Canton of Geneva. The study was conducted according to the Declaration of Helsinki.

All patients were recruited from the Memory Clinic of Geneva University Hospital. They had a diagnosis of Mild Cognitive Impairment (MCI) or early dementia due to probable Alzheimer’s disease (AD) based on clinical assessment. All had a positive amyloid positron emission tomography (PET) scan, and other neuroimaging signs of AD pathology: hippocampal atrophy or/and pathological values of fluorodeoxyglucose PET scan. Table 1 summarizes demographic data.

**Table 1:** Demographic and neuropsychological data

	Patients	Controls	
	n = 24	n = 19	
Age	72,0 ± 6,0	69 ± 7,0	n.s.
Years of education	12,5 ± 3,3	19 ± 6,0	*
Gender			
Women	*N* = 16(57%)	*N* = 9(47%)	
Men	*N* = 12(43%)	*N* = 10(53%)	
Orientation			
Spatial	4,0 ± 0,7		
Personal	4,8 ± 0,5		
Circonstantial	4,6 ± 0,6		
Temporal	3,7 ± 1,4		
Total orientation	17,2 ± 2,4		
MMSE			
Total score	23,6 ± 3,9	28,4 ± 1,6	*
Orientation	7,7 ± 1,9	9,6 ± 0,6	*
Recollection 3 words	1,1 ± 1,1	2,5 ± 0,8	*
Memory task			
First immediate recall	13,3 ± 3,2		
First free recall	4,0 ± 2,4		
First cued recall	5,1 ± 2,9		
Digit span			
Direct	8,2 ± 1,9	9,4 ± 2,2	n.s.
Indirect	6,2 ± 1,7	6,9 ± 1,9	n.s.

*MMSE* mini mental state examination; *n.s.* non significant; *significant differences (*p* < 0.05)

Nineteen healthy, age-matched controls with no history of neurological or psychiatric illness, and no signs of cognitive decline were recruited in the community through word of mouth and announcements in various Geneva senior associations. These subjects underwent the MMSE and Digit Span tests during the experimental task with EEG session. Their MMSE score was 28.4 ± 1.6, conforming to unimpaired cognitive functioning (Korsnes, 2020).

### Experimental Paradigm

**Fig. 1:**
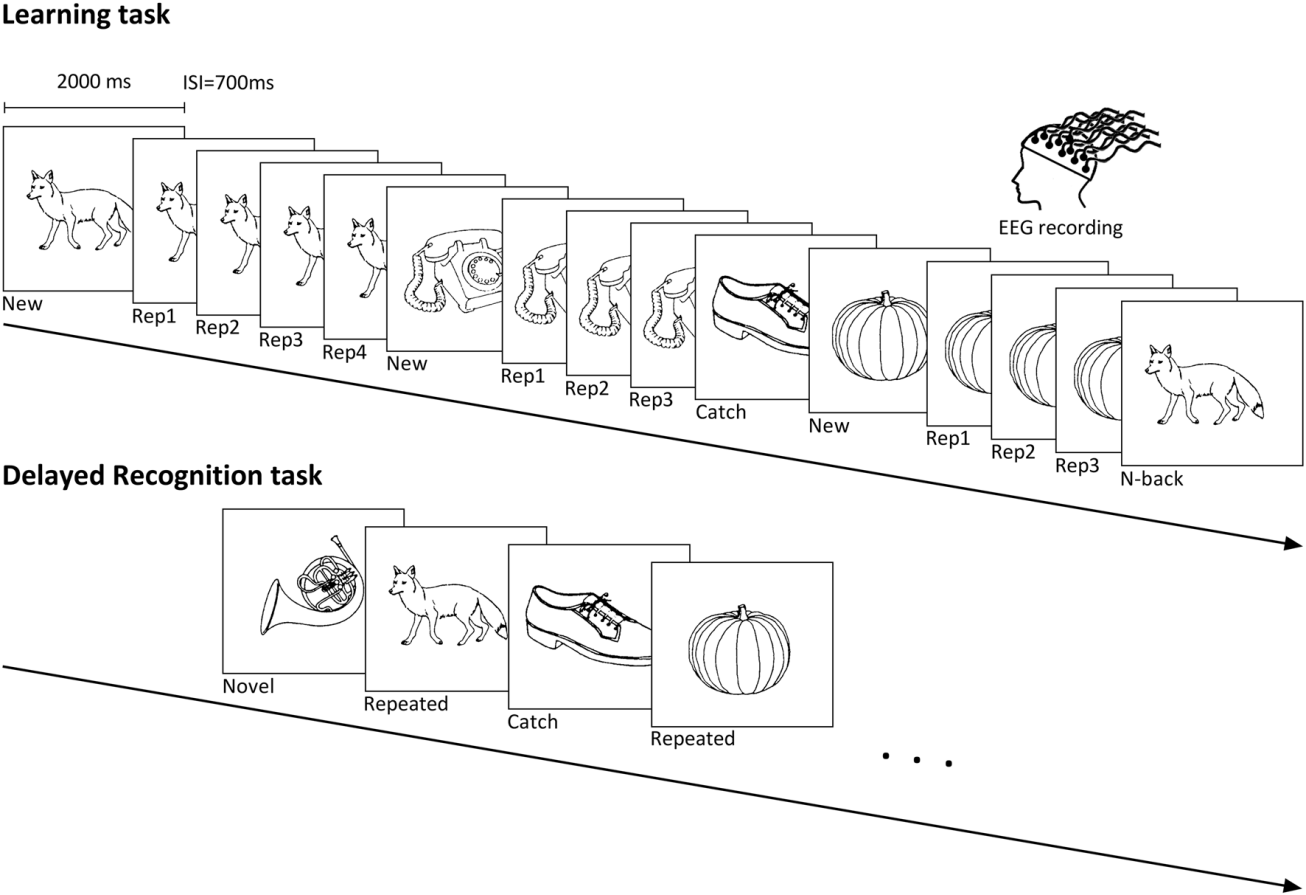
Continuous recognition task design. The learning task consists of two blocks containing different set of pictures. Subjects had to indicate if the presented picture is new (New) or repeated. Pictures were repeated up to four consecutive times (Rep1, Rep2, Rep3, Rep4) and then re-appeared after 9–15 intervening items (N-Back). Pictures presented only once in the sequence (Catch) were inserted in order to avoid the habituation to the sequence. To test recollection capacity, 30 min later pictures, repeated several times in the learning task (Repeated), Catch, and new pictures (Novel) were presented only once and subjects had to indicate their recurrence

Subjects performed a continuous picture recognition task (CRT), composed of concrete black and white line drawings from Snodgrass and Vanderwart (1980) (Fig. 1). Subjects had to indicate picture recurrences. Healthy participants responded by pressing the right button of the response box with their right middle finger if they had already seen the picture appearing on the screen, and the left button with their index finger if they had not seen the stimulus before. To prevent confusion of response keys and difficulties in multi-tasking, patients were asked to indicate their response verbally to the examiner, who pressed the corresponding button on the response box. Stimuli were presented on a white computer screen until the response, with a 700 ms inter-stimulus interval. If response time was longer than 2000 ms, picture would be exchanged by a white screen until the response button was pressed.

#### Main Learning Task

Participants firstly performed two blocks of a learning task (LT) separated by a 5 min’ break. Each block contained New pictures (*New*, *N* = 72) which were immediately repeated up to 4 consecutive times (*Rep1, Rep2, Rep3* trials, each *N* = 72, *Rep4* trial, *N* = 36). Pictures then re-appeared after 9–15 intervening items (*N-back*, *N* = 66). 22 pictures were presented only once and served as Catch trials to avoid the routine and give the impression that not all new the stimuli were repeated: they were presented randomly after Rep3, Rep4 or N-back stimuli. To avoid the expectation of a new picture after 3 repetitions, 36 pictures were repeated 4 times (*Rep4* stimuli). Thus, in total, there were 72 New, 72 Rep3 and 66 N-back stimuli.

The interest of the stimulus types was as follows: (1) New pictures represent novelty and undergo encoding. (2) N-back pictures are not novel, but they undergo re-encoding, as indicated by the fact that they are better recognized after a delay (e.g. 30 min) than stimuli presented only once (James et al., 2009). (3) Rep3 pictures are highly familiar after 3 consecutive presentations. Such repetitions induce repetition suppression, that is, reduction of neural activity, pronounced in the MTL (Grill-Spector et al., 2006; Henson et al., 2003; Johnson et al., 2008; Yassa & Stark, 2008). Thus, these stimuli provide no novelty and induce no or minimal encoding. They thus also serve as baseline condition to determine encoding effects with the other stimuli.

#### Delayed Recognition Task

To test long-term retention, subjects performed a delayed recognition task (DRT) 30 min later, which consisted of pictures presented repeatedly (*N* = 60) or as Catch trials (*N* = 22) in the learning task, plus new stimuli never presented before (*Novel*, *N* = 30). Participants were asked to indicate whether the presented picture was new or previously seen in the learning task.

### Data Acquisition

EEG was recorded continuously at 512 Hz using the 128 channels Active-Two Biosemi EEG system (BioSemi Active-Two, V.O.F., Amsterdam, The Netherlands). EEG data pre-processing and analyses were performed using the CarTool software (Brunet et al. (2011), https://sites.google.com/site/fbmlab/cartool), MATLAB 2012a, and the Statistical Toolbox for Electrical Neuroimaging (STEN), developed by Jean-François Knebel and Michael Notter (10.5281/zenodo.1164038). Offline, the EEG data was band-pass filtered to 1–30 Hz applying the second order Butterworth low and high pass filters, with -12 db/octave roll-off, recalculated against the average reference, and smoothened spatially using the instantaneous spatial filter (Michel & Brunet, 2019). Epochs from 100 ms before stimulus onset to 450 ms post-stimulus onset were averaged for each subject and for each stimulus type of the LT (New, R3, and N-back items) to compute event-related potentials (ERPs). Only trials with correct responses were selected for analyses. Eye blinks and cardiac artifacts were removed using Independent Component Analyses (ICA) based on the time course of the ICA component and its topography. Defective electrodes were interpolated using 3D spline interpolation (Perrin et al., 1987). Finally, each trial was visually inspected and epochs with remaining noise were excluded from analyses. The average number of accepted epochs was similar between and within groups for each condition (repeated measures ANOVA, all *p* > 0.05), and were as follows: mean ± SD; Healthy subjects: New, 57 ± 5; Rep3, 57 ± 4; N-back, 56 ± 6; Patients: New, 55 ± 6; Rep3, 56 ± 3; N-back, 54 ± 7.

### Behavioural Data Analysis

Demographic and behavioural data were analysed using SPSS, version 20. Performance in LT and DRT was determined as the sum of hits (correct recognition of repeated stimuli) and correct rejections of non-repeated stimuli. Repeated measures analysis of variance (rmANOVA) were performed on the percentage of correct trials with factors Group (Patients, Controls) and Condition (New, Rep3, and N-back in the LT, and Novel, Repeated and Catch in the DRT) separately for the LT and the DRT. In case of violation to the assumption of sphericity, Greenhouse–Geisser correction was used. Effect sizes are reported with the partial eta square (ɳ_p_^2^). Post-hoc pairwise comparisons were performed when significant effects were found with Bonferroni correction for multiple comparisons.

To further investigate relations between subjects’ performance in our CRT and clinical variables that best describe the two groups, we performed correlational analyses between MMSE total score, orientation and recollection scores and response accuracy across experimental conditions. Partial correlations accounting for subjects’ age and years of education were computed separately in Controls and Patients groups.

### ERP Waveform Analysis

EEG analyses were performed only on stimuli from the learning task. For determination of periods with effects over the whole set of electrodes, we computed electrode and time-wise ANOVAs on amplitudes in response to all 3 experimental stimulus types (New, R3 and N-back) for each of the 128 electrodes in Controls and Patients groups. To correct for temporal autocorrelation, only amplitude differences extending over at least 20 ms (10 time points) with *p* < 0.01, in the cluster of minimum 6 neighbouring electrodes, were retained (Murray et al., 2006).

### Peak Amplitudes Analyses

To investigate the direction of amplitude effects we further extracted ERP waveforms over 3 scalp regions of interests (ROIs): frontal (12 channels: C12, C13, C14, AF4, C18, AFz, C20, Fz, F1, C26, C27, AF3), central (12 channels: Cz, A2, CPz, B1, CP2, C1, C2, FCz, D1, D2, D15, D16) and parietal (12 channels: O1, A15, A16, PO3, A21, A22, Oz, A24, A27, O2, A29, A30) clusters corresponding to the International 10–20 system (Jurcak et al., 2007). Since the latency of ERP components might be shifted between and within groups, we performed amplitudes’ peak analyses. The time periods of peak amplitudes were chosen based on the observation of grand-averaged ERP waveforms over frontal, central and parietal clusters in Control and Patients groups, and were as follows: In the Frontal and Central ROIs, most positive peaks at 150–250 ms after stimulus onset and the most negative peaks at 225–360 ms; in the parietal cluster, most negative peaks at 140–210 ms and the most positive peaks at 225–400 ms. Peak amplitudes in these time windows were extracted for each condition and ROI at the single subject level. They were compared statistically with 2 (group: Controls and Patients) × 3 (condition: New, Rep3 and N-back) repeated measures ANOVA (rmANOVA) separately for each ROI and ERP component. In cases where sphericity was violated Greenhouse–Geisser correction was applied. We report the effect sizes with the partial eta square (*ɳ*_p_^2^).

### Source Estimation

The localization of the neural generators underlying the ERPs of each experimental condition were estimated using the distributed linear inverse solution model of the low resolution brain electromagnetic tomography (LORETA) (de Peralta Menendez et al., 2001; de Peralta Menendez et al., 2004). For Controls the current distribution was estimated from 128 scalp electrodes within the grey matter of the Montreal Neurological Institute (MNI) template using the LSMAC head model adapting the scalp conductivity (Hoekema et al., 2003; Latikka et al., 2001) to the mean age of our sample (69 years old) with a solution space of 5000 nodes (Brunet et al., 2011).

In the Patients, source localization was performed using individual MRI scans. Each patient’s structural MRI was segmented with Cartool software that allowed to extract the grey matter and to obtain the full model of the head (the scalp, the skull, and white and gray matters). Electrodes’ position was co-registered on each subject’s scalp. The 5000 solution points were selected within the extracted grey matter, and the lead field with the LSMAC model was calculated by adapting the scalp conductivity with each subject’s age using Cartool. One subject in the Patients group was eliminated from this analysis due to bad MRI imaging data.

To avoid spatial leakage and activation biases each solution point was standardized across time (Michel & Brunet, 2019). The estimated current densities of inverse solution points were then extracted and averaged per participant and condition in the periods showing significant main effects of Condition in the waveform analyses. The averaged signal over time periods of interest was then compared statistically between experimental conditions with rmANOVA with Group as between-subjects factor, and Condition as within-subjects factor. Only effects significant at *p* < 0.01 and with clusters of minimum 20 nodes were retained (Knebel & Murray, 2012).

## Results

### Behavioural Results

Behavioral results are summarized in Table 1. Age was similar in both groups; years of education were significantly higher in Controls (19 ± 6 years) than Patients (12.5 ± 3.3 years). Compared to Controls, Patients had significantly lower total MMSE score, and lower scores in two MMSE subdomains: orientation and recall of three words. Digit span did not significantly differ between the groups.

### Performance in the Tasks

#### Learning Task

**Fig. 2:**
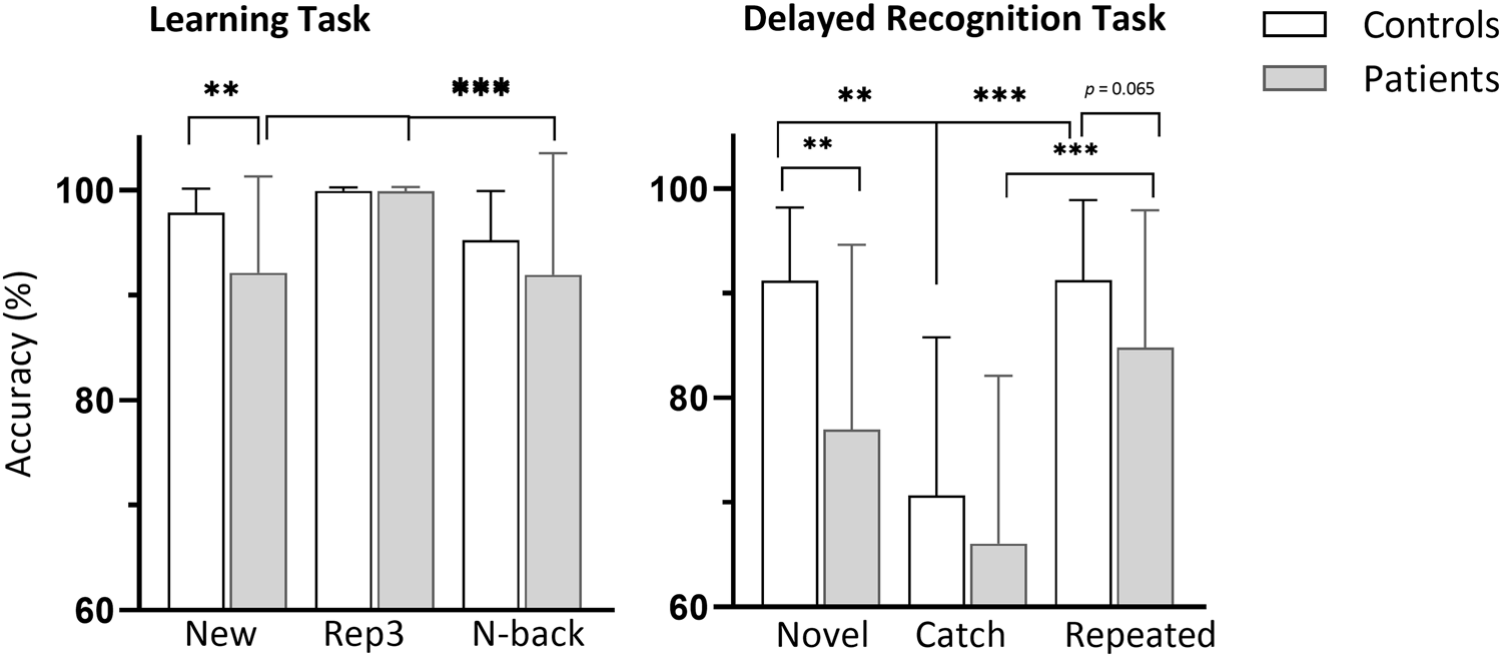
Subjects’ performance in experimental task. Error bars show standard error of the mean. **p < 0.01, ***p < 0.005

Response accuracy is shown in Fig. 2. A repeated-measures ANOVA with Group and Condition (New, Rep3, N-back) revealed significant main effects of Condition (F _(1.44, 59.06)_ = 9.77, *p* = 0.001, *ɳ*_p_^2^ = 0.192) and Group (*F*_(1, 41)_ = 7.48, *p* = 0.009, *ɳ*_p_^2^ = 0.154) in the LT. Accuracy was higher in Controls than Patients (*p* = 0.009). The group effect was essentially driven by worse performance on New items in Patients than Controls (*p* = 0.011): Patients incorrectly endorsed almost 8% of New items as seen before, Controls only 2%.

Patients recognized Rep3 items more accurately (99.8 ± 0.39%) than New (92.1 ± 9.1%, *p* < 0.005) and N-back items (91.2 ± 11.6%, *p* < 0.005). In Controls, response accuracy did not differ significantly between stimuli (New: 97.8 ± 2.28%; Rep3: 99.9 ± 0.32%; N-back: 95.2 ± 4.69%). The interaction between group and condition did not reach significance.

Response latencies in Controls were the longest in response to Catch (1002 ± 253), N-back (998 ± 179), and New stimuli (995 ± 136), and decreased gradually with each immediate consecutive repetition (Rep1: 739 ± 138; Rep2: 582 ± 126; Rep3: 536 ± 145; Rep4: 531 ± 117). The Rep3 were recognized faster (rmANOVA, *F*_(2, 36)_ = 147.88, *p* < 0.005, *ɳ*_p_^2^ = 0.891) than New and N-back pictures, confirming the high familiarity effect elicited by Rep3 items.

#### Delayed Recognition Task

There were significant main effects of Condition (F _(1.33, 54.87)_ = 22.13, *p* < 0.005, *ɳ*_p_^2^ = 0.351) and Group (F _(1, 41)_ = 15.22, *p* < 0.005, *ɳ*_p_^2^ = 0.271). The interaction between group and condition was not significant. Overall, Controls performed better than Patients (*p* < 0.001). The group effect was again due to worse performance of Patients in correctly rejecting novel stimuli than Controls (false positives: Patients 23%; vs Controls 9%). Catch items, which were presented only once in the LT, were recognized worse (Controls: 70,6 ± 15,1; Patients: 66,1 ± 16) than Novel (91.2 ± 6.9, *p* < 0.005) and Repeated (91.3 ± 7.6, *p* < 0.01) items by Controls and worse than Repeated items (84.7 ± 13.1, *p* < 0.005) by the Patients.

#### Correlations

In Patients, there was a positive correlation between response accuracy to New items and the MMSE total score (*r* = 0.428, *p* < 0.05) and the MMSE recollection score (*r* = 0.462, *p* < 0.05). Correct rejections of New items in the LT correlated positively with response accuracy to Novel and Catch items in DRT (*r* = 0.425, *p* < 0.05, *r* = 0.739, *p* < 0.005 respectively). Hits to N-back items in LT correlated with hits to repeated items in DRT (*r* = 0.54, *p* < 0.05). In Controls only hits to N-back in the LT correlated significantly with the MMSE recollection score (*r* = 0.616, *p* < 0.01).

### ERP Waveform Analysis

**Fig. 3:**
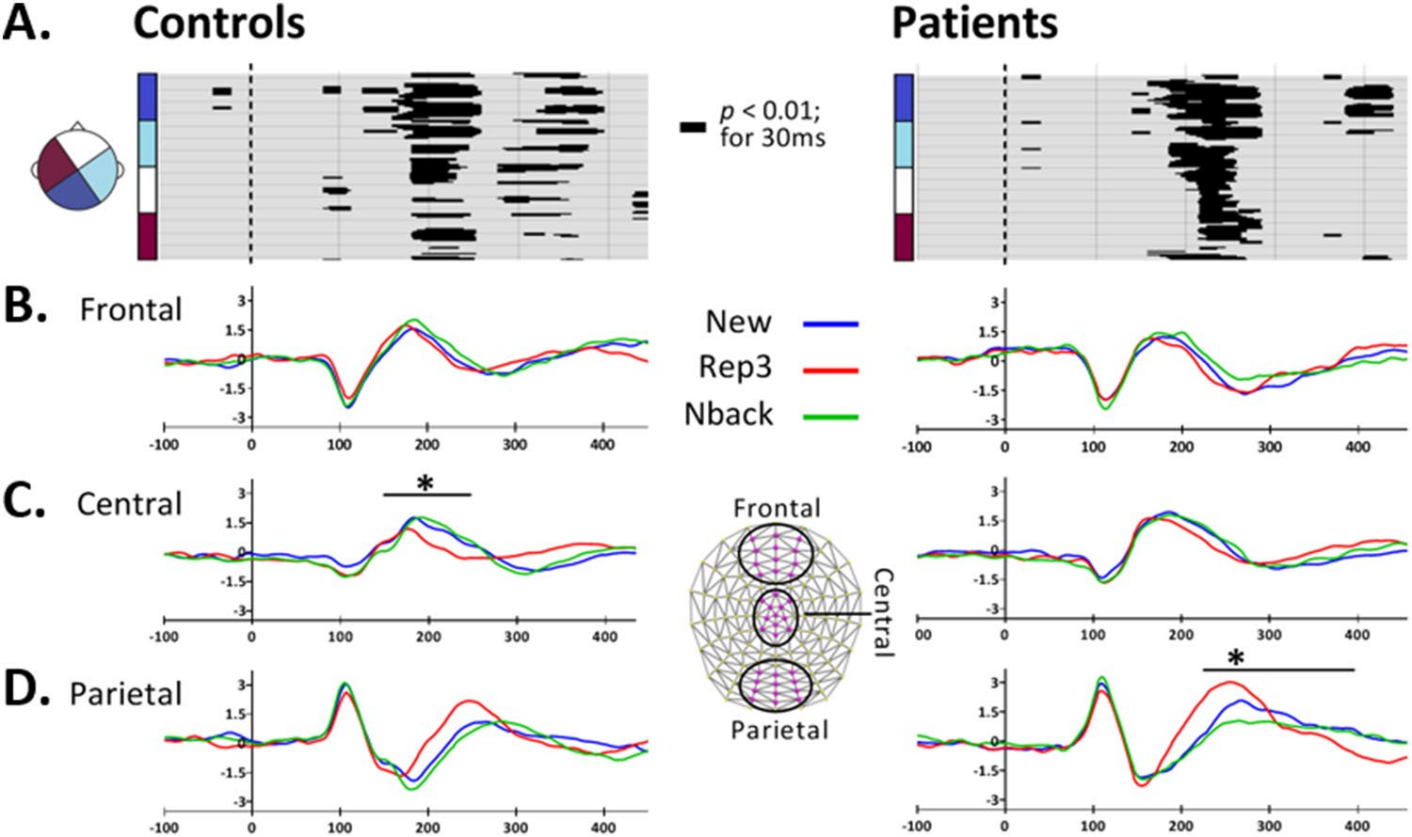
ERP waveform analysis. **A** Amplitude differences over all electrodes across conditions revealed by one-way ANOVAs in controls and patients. The y axes display the 128 electrodes. **B**–**D** Waveforms on Frontal (**B)**, Central (**C**) and Parietal (**D**) clusters. The vertical axes indicate the amplitude (in *µV*) The asterisks show significant differences between peak amplitudes; the line shows the time period where the peak analyses was performed. Amplitude peak analysis was performed for positive peaks at 150–250 ms and negative peaks at 225–360 ms over frontal and central clusters, and negative peaks at 140–210 ms and positive peaks at 225–400 ms over parietal clusters

The effects retained with time-wise one-way ANOVAs (*p* < 0.01, ≥ 20 ms; ≥ 6 neighboring electrodes) on ERP amplitudes over all scalp electrodes are displayed in Fig. 3A. In Controls, a main effect of condition was observed at 180–260 ms post-stimulus onset over extended scalp regions, and at around 285–400 ms following stimulus onset on fewer electrodes over varied scalp areas. In Patients, the main condition effect fell at around 180–280 ms after stimulus onset over extensive scalp areas, and brief effects on few electrodes at 380–430 ms.

### ERP Peak Analysis

**Fig. 4:**
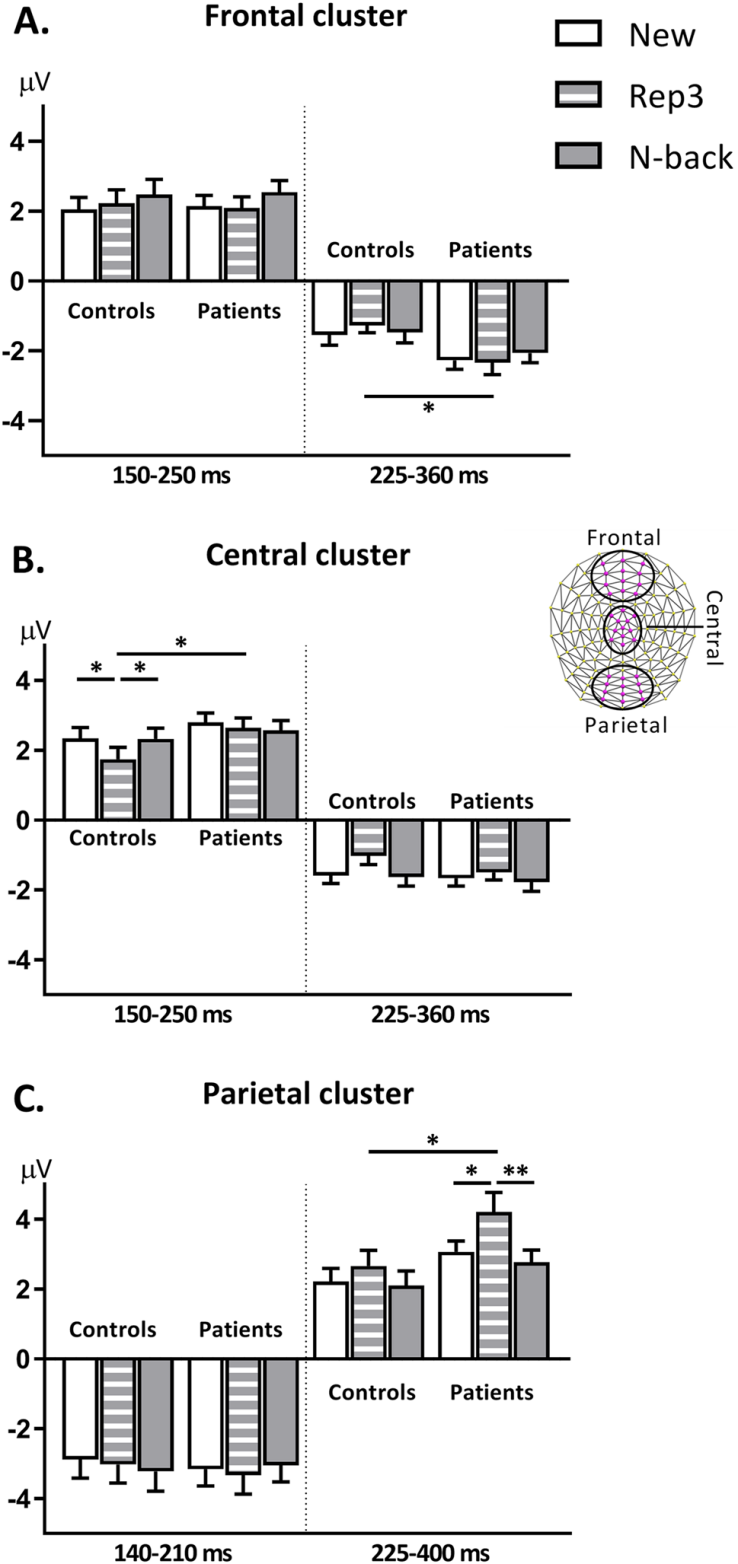
ERP peak analysis. Results of repeated-measures ANOVA on amplitude peaks over **A** Frontal, **B** Central and **C** Parietal clusters between controls and patients. Error bars show standard error of the mean. *p < 0.05, **p < 0.01, ***p < 0.005

ERP waveforms on the frontal, central and parietal ROIs are shown in Fig. 3B-D. This illustration suggests that differences observed in the general waveform analysis might partly reflect temporal shifts of waves, in particular accelerated processing of R3 stimuli, as also indicated by the faster reaction times in controls. Figure 4 depicts comparison of peak amplitudes, independent of the time of peak, within and between the two groups in frontal, central and parietal ROIs (Fig. 4 A, B and C respectively).

#### Frontal Cluster

rmANOVA on positive peaks at 150–250 ms after stimulus onset yielded no significant Group x Condition interaction or group effect. There was a significant main effect of condition (*F*_(1.71, 70.09)_ = 3.99, *p* = 0.029, *ɳ*_p_^2^ = 0.08), driven by overall peak differences between N-back and New (*p* = 0.019) and Rep3 (*p* = 0.045). This effect did not differ significantly within each group. Negative peaks at 225–360 ms significantly differed between groups (*F*_(1, 41)_ = 5.44, *p* = 0.025, *ɳ*_p_^2^ = 0.117), with Rep3 peaks being more negative in Patients than Controls (*p* = 0.017).

#### Central Cluster

Analyses on the positive peaks at 150–250 ms in the central ROI revealed no significant group x condition interaction or main effect of group. There was a significant main effect of condition (*F*_(1.56, 63.84)_ = 4.49, *p* = 0.022, *ɳ*_p_^2^ = 0.099) driven by less positive Rep3 amplitude as compared to New (*p* = 0.021) and N-back (*p* = 0.037) in the Control group. At 225–360 ms, there was a significant main effect of condition (*F*_(1.64, 67.61)_ = 4.34, *p* = 0.023, *ɳ*_p_^2^ = 0.096).

#### Parietal Cluster

No significant effects were found on negative peaks at 140–210 ms. Analyses in the time window of 225–400 ms on positive peaks revealed a significant main effect of condition (*F*_(1.42, 58.39)_ = 9.11, *p* = 0.001, *ɳ*_p_^2^ = 0.18) due to more positive Rep3 response peaks in comparison to N-back (*p* = 0.001) and New (*p* = 0.018) in the Patient group. Patients expressed a higher response to Rep3 than Controls (*p* = 0.044).

### Source Estimation

**Fig. 5:**
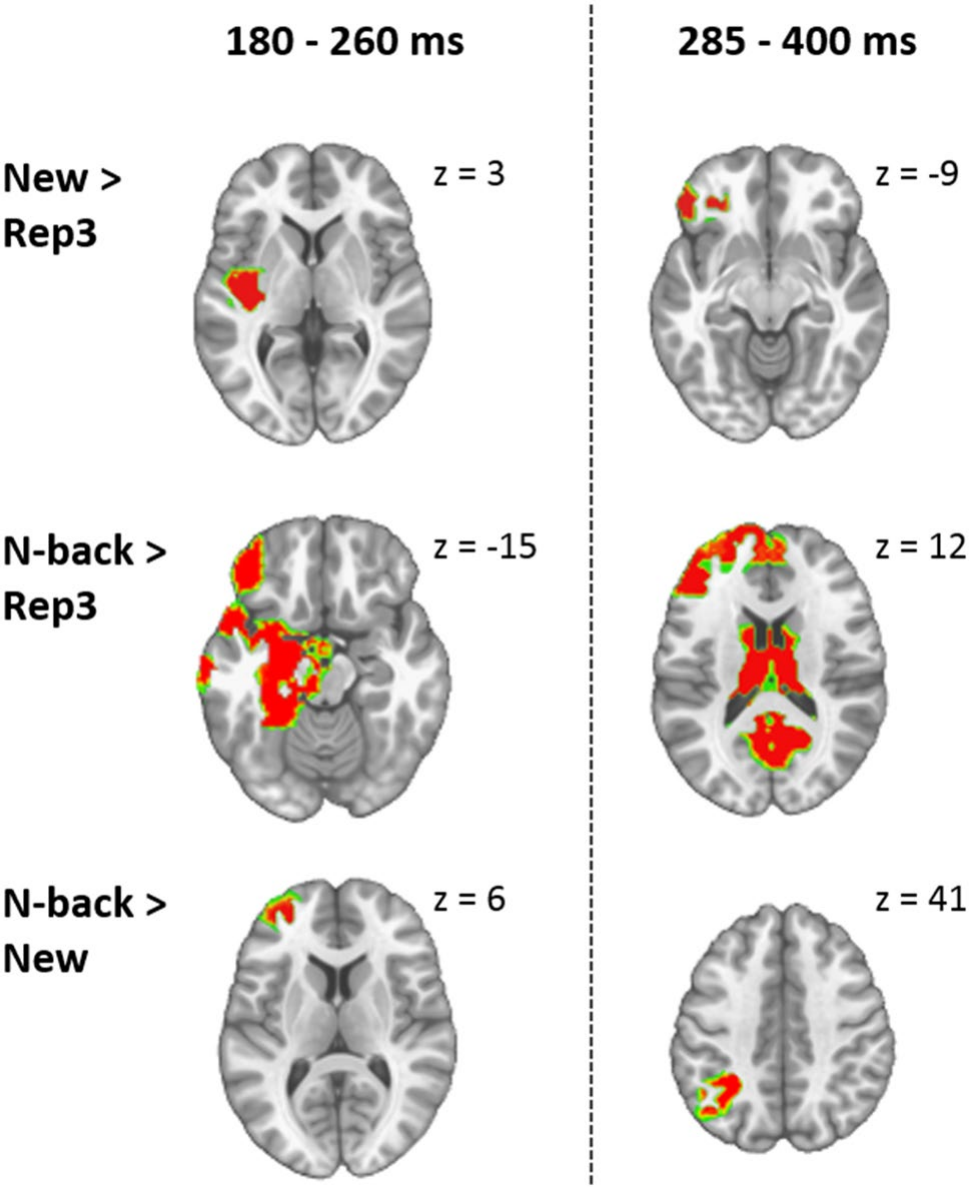
Neural source estimations in controls. Figure displays the *post-hoc* paired *T*-test (with *p* < 0.01, controlling for cluster of 20 contiguous nodes) comparing New, Rep3 and N-back conditions in two periods of interest: 180–260 ms and 285–400 ms

Within-group analysis: Fig. 5 displays *post-hoc* paired *T*-test (*p* < 0.01; with 20 adjacent voxels) on solution points averaged over the two periods of interest (180–260 ms and 280–400 ms).

In the Patient group, there were no significant differences between conditions in either period of interest, suggesting similar generators of brain activity in response to all 3 stimulus types.

In Controls source estimations revealed that in the early period (180–260 ms) New compared to Rep3 items more strongly activated left middle and superior gyri in the anterior part of the temporal lobe (Fig. 5). N-Back in comparison to Rep3 items induced greater activation in extended left MTL region: left hippocampus, the anterior part of left temporal lobe, left inferior and superior temporal gyri, thalamus, and other areas: left insula, middle-frontal gyrus and superior medial frontal gyrus (Fig. 5). N-Back items more strongly than New items activated a small area in the middle frontal gyrus (Fig. 5).

In the later period (280–400 ms), New compared to Rep3 stimuli more strongly activated the inferior frontal gyrus. N-back in comparison to Rep3 induced stronger activations in left middle frontal gyrus, bilateral superior frontal gyri, left hippocampus, the thalamus and cerebellum. N-back stimuli as compared to New induced greater activations in left angular gyrus.

#### Between Group Comparisons

**Fig. 6:**
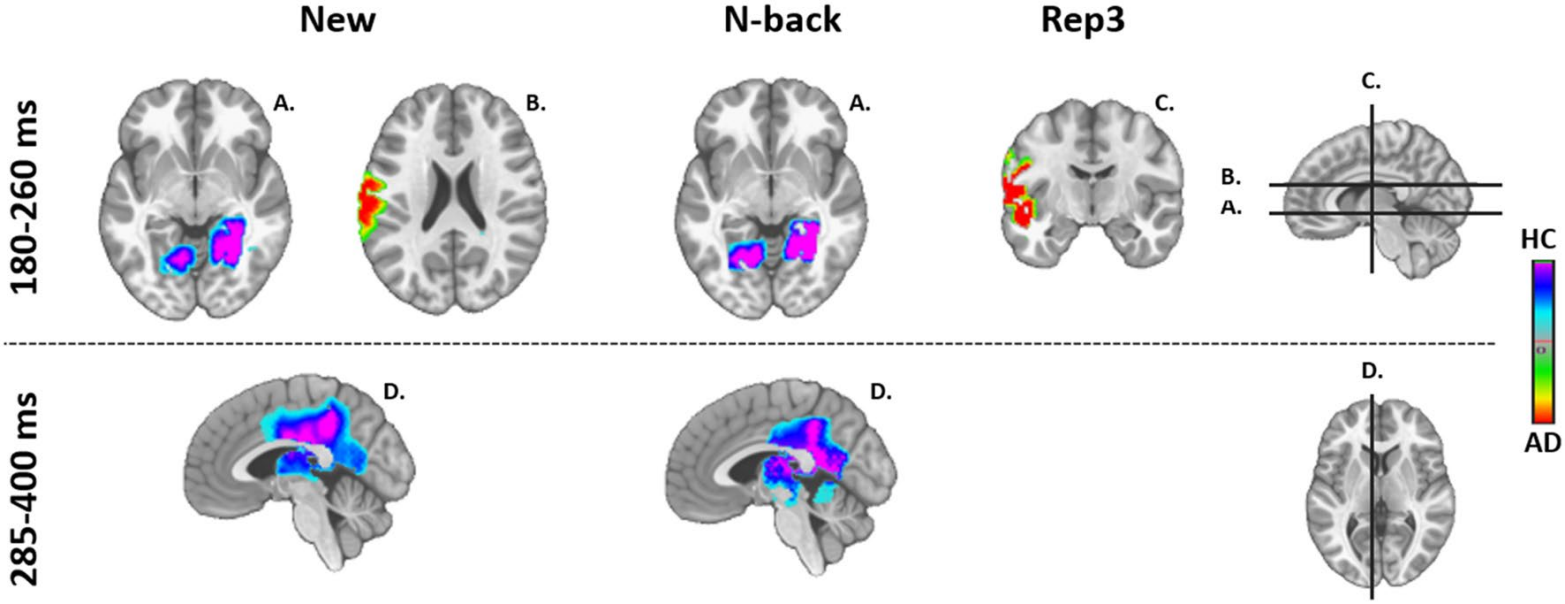
Comparison of reconstructed sources between groups. Figure shows the results of *post-hoc* unpaired *T*-tests (with *p* < 0.01, controlling for cluster of 20 contiguous nodes) in two periods of interest (180–260 ms and 285–400 ms). The scale indicates colour meanings. HC, healthy controls; AD, Alzheimer’s disease patients. **A**, **B**, **C** and **D** letters show the level at which different brain sections are displayed

Fig. 6 shows the comparisons between groups. In the early time window (180–260 ms) New stimuli more strongly activated the right posterior hippocampus, medial and inferior parts of occipital lobe and some areas in left cerebellum in Controls than in Patients. Conversely, in Patients, they more strongly activated the left post-central gyrus and superior temporal gyrus. N-back stimuli more strongly activated the right posterior hippocampus, right precuneus, left thalamus and medial and inferior areas in occipital lobe in Controls than Patients. Rep3 stimuli induced stronger activations in left superior temporal gyrus and left medial superior frontal gyrus in Patients as compared to Controls.

In the later time window (285–400 ms) Controls in comparison to Patients activated more the thalamus and middle cingulate gyrus in response to New stimuli, and middle and posterior cingulate gyrus in response to N-back stimuli. Rep3 activations did not differ between groups in this time window.

## Discussion

Patients with early AD, in comparison to age-matched controls, presented a deficit in novelty detection and encoding, reflected in significant early differences in electrocortical activity in the first 400 ms after stimulus presentation. While differences in peak amplitudes between groups were relatively small, analysis of the cortical generators of activity using inverse solution revealed strikingly decreased activation of the MTL and extended cortical networks in AD patients. Activations appeared to be driven by encoding activity with no additional activations attributable to novelty.

A potential caveat of our study lies in the fact that patients responded verbally, while controls responded by pressing a button. Given that the ERP effects occurred very early, well before motor reactions (of controls), but also considering the MTL activations observed, we think that the results reflect novelty and memory processing with no interference by response modality.

Both in the learning and delayed recognition task, patients failed to discriminate new from old stimuli; they had an increased false alarm rate. The severity of this failure correlated with the total score and the recollection score (the 3 words) in the MMSE. At first sight, this result would be compatible with a response bias to say “yes” to any stimulus. Such a bias was previously observed in AD patients (Budson et al., 2000; Pillon et al., 1993) and might be explained by frontal executive dysfunction (Baddeley et al., 2001). In our patients, however, this explanation appears unlikely because they also failed to normally recognize picture recurrences, that is, they also had a low hit rate in both tasks. A possible interpretation of the increased false positive rate might be that, due to inefficient encoding our patients relied on overly broad gist memory (Schacter et al., 1998) rather than on item-specific recollection. Regardless of the underlying mechanism, these findings demonstrate deficient novelty detection and impaired encoding in our group of patients with early AD.

Throughout the analyses of the EEG, New and N-back stimuli (repetitions after intervening items), evoked very similar responses, while both differed from the Rep3 stimuli (presentations after 3 immediate repetitions). In the waveform analyses, there were two early time periods of amplitude differences between the stimuli, with most significant effects occurring at 180–260 ms in controls and patients, and to a lesser extent at 280–400 ms after stimulus onset in controls. These time periods largely overlap with findings of a previous study using a similar paradigm, which showed MTL-mediated responses to new stimuli, in comparison to highly familiar stimuli, at 200–300 ms in healthy subjects (Raynal et al., 2020). In the present study, controls had higher peak amplitudes when processing New and N-back items than Rep3 pictures over central electrodes at 150–250 ms. The time range of this peak corresponds to the positive fronto-central component (P2) which is believed to signify modulations of perceptual processing, attention (Luck & Hillyard, 1994) or working memory (Lefebvre et al., 2005). A stronger positivity in response to New and N-back than Rep3 items is therefore compatible with encoding-related processing, which requires recruitment of attention and working memory. Interestingly, similarly to a previous study (Li et al., 2016) patients did not express this potential difference at central electrodes, suggesting impaired processing of new items or items repeated after several intervening stimuli.

The most obvious ERP difference in patients occurred over parietal electrodes (Fig. 3D), where Rep3 induced a more positive peak amplitude than New and N-back items at 225–400 ms. The time-range of this peak corresponds to the P300 component, thought to reflect stimulus relevance and novelty (Azizian & Polich, 2007; Patel & Azzam, 2005; Polich, 2007; Sur & Sinha, 2009). Studies using the oddball paradigm have shown an enhanced P300 in response to novel stimuli (Ranganath & Rainer, 2003). In AD patients, the P300 amplitude is typically attenuated and its latency is increased (Hedges et al., 2016; Olichney et al., 2011). In contrast to the oddball task, our paradigm requires differentiating between new and repeated items, rather than detecting an outstanding stimulus. As such, this increased amplitude in our patients might reflect more attentional effort required to process massed item repetitions. This is the contrary of what would be expected from the processing of repeated stimuli, namely facilitated processing.

Modulations in waveform amplitudes do not necessarily indicate different neural generators (Murray et al., 2008). In this study neural source estimations revealed stronger differences in the involved brain regions than expected on the basis of the relatively subtle differences observed in the waveforms. At 180–260 ms, controls activated wider regions when processing New and N-back than Rep3 items (Fig. 5): MTL, thalamus and some frontal areas in response to N-back items, anterior part of left temporal lobe in response to New items. New and N-back items generated almost indistinguishable activity, suggesting that novelty does not induce more wide-spread brain activity than already seen pictures. Rep3 did not activate any region beyond the other stimuli. These activations are coherent with previous studies showing that the hippocampus and other structures within the MTL are essential for processing novel information (de Chastelaine et al., 2017; Köhler et al., 2005; Kumaran & Maguire, 2009; Ranganath & Rainer, 2003; Strange et al., 2005) and for encoding (Habib et al., 2003; Stern et al., 1996; Tulving et al., 1996). MTL activity related to encoding was even shown to predict enhanced subsequent recognition (Habib et al., 2003; Kirchhoff et al., 2000). Based on these findings it is remarkable that source estimations in patients revealed no activation differences between the three stimulus types in this early time window. This suggests less distinguishable processing of novel and repeated stimuli in patients by similar underlying neural generators.

In the subsequent period, at 285–400 ms, amplitudes in the waveforms differed over various scalp regions between stimulus types in controls, but, except for some short differences on few parietal electrodes, not in patients (Fig. 3A). Similarly, source estimations showed strong inter-stimulus differences in controls, but not in patients. While new stimuli induced stronger activation than Rep3 in a restricted lateral frontal area, N-back induced stronger activations in a large area including frontal regions, left hippocampus, thalamus and cerebellum. These activations might reflect an ongoing stimulus appraisal and evaluation process. As argued by Kafkas and Montaldi 2018, the thalamus might mediate integration of information about the stimulus, by combining MTL-sensible novelty signals with familiarity-related information from prefrontal cortex.

Thus, in striking contrast to controls, patients failed in both periods to activate different brain regions in response to the three stimulus types. While this may be due to a failure to activate structures critical for encoding, novelty detection, or both, an alternative interpretation might be that patients fail to appreciate the multiple repetitions leading up to the Rep3 stimulus presentation. Repetition suppression – the decrease of neural activity to repeated presentation of regions specialized for a specific stimulus attribute – occurs in occipito-temporal cortex and the MTL (Grill-Spector et al., 2006; Henson et al., 2003; Yassa & Stark, 2008). In MCI and AD patients, it has been found to be impaired (Golby et al., 2005; Pihlajamäki et al., 2008, 2011), which is also in line with the increased ERP amplitudes associated with Rep3 stimuli in our AD patients.

While the analysis of Rep3 suggests impaired, or even absent, repetition suppression the direct comparison between groups also provides clear evidence that AD patients fail to normally activate MTL and paralimbic areas when processing New and N-back items. In the early period of 180–260 ms, controls more strongly activated the MTL in response to New and N-back stimuli than AD patients (Fig. 6). In patients, superior activity occurred in response to all 3 stimulus types in left temporo-parietal cortex. Given the importance of the MTL for processing novelty (de Chastelaine et al., 2017; Köhler et al., 2005; Kumaran & Maguire, 2009; Ranganath & Rainer, 2003; Strange et al., 2005) and encoding (Habib et al., 2003; Kirchhoff et al., 2000; Stern et al., 1996; Tulving et al., 1996), this lack of early activation of the MTL may explain the memory deficits characteristic of beginning AD. Previous fMRI studies appeared to contradict our observations: they showed enhanced MTL activation in early AD patients during the encoding of words or picture-word pairs (Hämäläinen et al., 2007; Kircher et al., 2007). Accordingly, MCI patients’ activations of the hippocampus plus posterior cingulate during encoding of words predicted better recognition (Papma et al., 2017). However, fMRI with its low temporal resolution probably also seizes activation reflecting the increased effort of the patients to explicitly store information. Conversely, our -effortless- continuous recognition paradigm and the brief period explored with EEG in this study likely reflect the automatic phase of novelty detection and encoding, not amenable to conscious compensation by effort.

In the second period, 285–400 ms, controls had greater activations in middle and posterior cingulate and the thalamus when processing New pictures or N-back items. The middle cingulate has been suggested to be involved in decision making (Apps et al., 2013; Bush et al., 2002) and attention control (Vogt, 2016). The posterior cingulate cortex is part of the default mode network, which preferentially activates at states of rest and deactivates during cognitive tasks demanding attention (Buckner, Andrews-Hanna, & Schacter, 2008; Leech & Sharp, 2014). It is also part of an episodic memory network which encompasses the MTL, retrosplenial cortex and prefrontal areas (Greicius et al., 2009). It was shown to be disrupted in patients with AD (Greicius et al., 2004). While the posterior cingulate is deactivated in most cognitive tasks (Buckner et al., 2008; Leech & Sharp, 2014), episodic memory tasks such as mnemonic search (Shapira-Lichter et al., 2013) or recognition of 2-back visuospatial stimuli as opposed to immediate repetitions (Carlson et al., 1998) appear to induce activation of this area. The present findings suggest dysfunction in the early phase of these processes in beginning AD.

Notwithstanding its clear results, our study has limitations. First, high-density EEG is capable of revealing activations of deep brain structures but does not have high spatial resolution. Thus, anatomical conclusions in this paper refer to regions (e.g., medial temporal area) rather than precise structures (e.g., hippocampus). Second, our results refer to patients with a clinical pattern of deficient memory and positive biomarkers for Alzheimer’s disease (amyloid, hippocampal atrophy, PET). But histologically defined AD is heterogeneous (Jellinger, 2021). It appears unlikely that patients with a non-amnestic variant of AD, e.g., posterior cortical atrophy or logopenic progressive aphasia, who lack the initial memory impairment observed in our patients (Whitwell et al., 2022), would present the same EEG alterations. It is also possible that non-AD amnesia (e.g., hypoxic encephalopathy) would induce similar results. Thus, our study reveals a principle of deficient memory processing in early AD, but with unknown specificity. Finally, as essential results of this study originated from sophisticated analyses and not from easily obtainable ERP waveforms, it would be difficult to apply our results as a marker of AD -or another form of amnesia- in clinical settings.

## Data Availability

Participants’ consent did not include public data sharing. Study data are available from the corresponding author upon reasonable request and on the condition of authorization by the Ethical Committee.
